# New Approach to Rare Pediatric Multicystic Mesenteric Lymphangioma; Would It Guide the Development of Targeted Therapy?

**DOI:** 10.3389/fped.2018.00223

**Published:** 2018-08-07

**Authors:** Rodica Heredea, Anca M. Cimpean, Simona Cerbu, Calin M. Popoiu, Adriana A. Jitariu, Marius Raica

**Affiliations:** ^1^Department of Pathology, “Louis Turcanu” Children's Clinical Emergency Hospital, Victor Babes University of Medicine and Pharmacy, Timisoara, Romania; ^2^Department of Microscopic Morphology/Histology, Victor Babes University of Medicine and Pharmacy, Timisoara, Romania; ^3^Angiogenesis Research Center, Victor Babes University of Medicine and Pharmacy, Timisoara, Romania; ^4^Department of Radiology, Victor Babes University of Medicine and Pharmacy, Timisoara, Romania; ^5^Department of Pediatric Surgery, Victor Babes University of Medicine and Pharmacy, Timisoara, Romania

**Keywords:** mesenteric cystic lymphangioma, podoplanin, Prox-1, PDGFRs, VEGFR-3

## Abstract

In children, lymphangiomas are extremely rare pathologic entities that are characterized by unusual locations. The mesenteric localization is extremely rare in children, and the clinical signs usually mimic an acute abdominal syndrome. For most of the cases, their diagnosis is established by the radiologist, and the main therapeutic option is represented by surgery for lesion removal. We hereby describe the case of a 4 year old girl admitted to the pediatric emergency department for continuous abdominal pain, more intense in the orthostatic position, associated with abdominal distension, nausea, and vomiting. These symptoms raised the clinical suspicion of acute abdominal syndrome. The patient had no previous clinically significant events. Radiologic examination suggested a mesenteric multicystic lymphangioma certified by surgical and histopathological evaluation. No specific targeted therapy is currently available; moreover, no specific criteria for recurrences have been stated. A new approach of infantile lymphangiomas following surgery, regarding the use of specific lymphatic markers panel including D2-40, Prox-1, VEGFR-3, PDGFs, and Ki67 may improve the characterization of such lesions regarding their prognosis, recurrence rate and targeted therapy implementation especially for those with a more aggressive or recurrent behavior.

## Introduction

Vascular tumors in children represent a challenge for both the pediatrician and the pathologist. Currently, no certified molecular classification is available, not even for more common vascular tumors such as congenital and infantile hemangiomas, and even less, a molecular characterization of other extremely rare vascular lesions that occur during childhood such as lymphangiomas with different locations. In children, lymphangiomas are extremely rare pathologic entities that are characterized by unusual locations. For most of the cases, their diagnosis is established by the radiologist, and the main therapeutic option is surgical removal of the lesion.

## Background

Usually, lymphangiomas localization in children interests the head and neck area (1), although isolated locations have been suggested at the level of the axillary zone (2) and only 10% in the mediastinum and abdominal cavity (3, 4). The most rare lymphangioma cases are encountered at the level of the retroperitoneal area (5). No matter their localization, surgery represented the gold standard for their treatment. There are cases reported in literature of recurrence, though most are attributed to incomplete excision, without other cellular, molecular or etiologic aspects taken into account (3–5).

The mesenteric localization is extremely rare in children, and the clinical signs usually mimic an acute abdominal syndrome. The first mesenteric lymphangioma ever to be subject to surgical excision dates back to 1802, the procedure being applied by Tillaux (6), followed by a detailed description made by Redenbacher in 1828 (7). In 1842, Rokitanski (7) also described a lymphangioma. Since their first description (1828), up to 2018, there were reported no more than 820 lymphangioma cases, amongst them only 72 being documented with an abdominal location, 6 of them being described as calcified. The fact that ~75% of lymphangioma lesions occur before birth and the remaining percentage in postnatal life, has raised questions regarding their origin and pathogenesis, thus leading to the emergence of numerous theories, starting from a series of genetic mutations to anatomical disorders during embryonic development.

Due to the extremely rare occurrence of such lesions during childhood, almost no characterization has been made from a molecular point of view, and moreover, drug-based therapy is limited. Some childhood lymphangiomas are easily misdiagnosed for hemangiomas and are improperly treated using propranolol (8). Their lack of response to this treatment imposes their reevaluation with a diagnostic switch to lymphangioma and application of surgery-based therapy. Sclerotherapy (9) as well as sirolimus-based therapy in case the lesion lacks response to propranolol (10), have been rarely approached until now. In any case, propranolol-based therapy seems to be inefficient in infantile lymphangiomas (11).

Targeted therapy represents the gold standard for several tumor conditions especially for those with malignant phenotype. This kind of therapy has several advantages to conventional ones because it acts specifically to tumor cells preserving the normal cells. It is well-known in that lymphatic endothelial cells (LECs) from tumors are different by molecular point of view compared with those from normal tissues. Prox-1 expression in LECs gives them an immature state. Immature LECs are more sensitive to targeted therapy compared to mature LECs because of their particular phenotype. Thus, specific therapies are needed to be developed. As a paradox, at the time being we do not dispose of a targeted therapy for specifically expressed markers of LECs, neither do we benefit from a large number of scientific studies that are focused on the characterization of lymphangiomas regarding LECs maturation markers as VEGFR-3, Prox-1, D2-40, PDGFR-β, or proliferation index such as Ki-67 (12, 13). Based on the above mentioned aspects, a detailed evaluation of infantile lymphangiomas by immunohistochemistry and/or molecular techniques, becomes mandatory.

## Case presentation

We hereby describe the case of a 4 year old girl admitted to the pediatric emergency department for continuous abdominal pain, more intense in the orthostatic position, associated with abdominal distension, nausea, and vomiting. These symptoms raised the clinical suspicion of acute abdominal syndrome. The patient had no previous clinically significant events.

**Figure 1:**
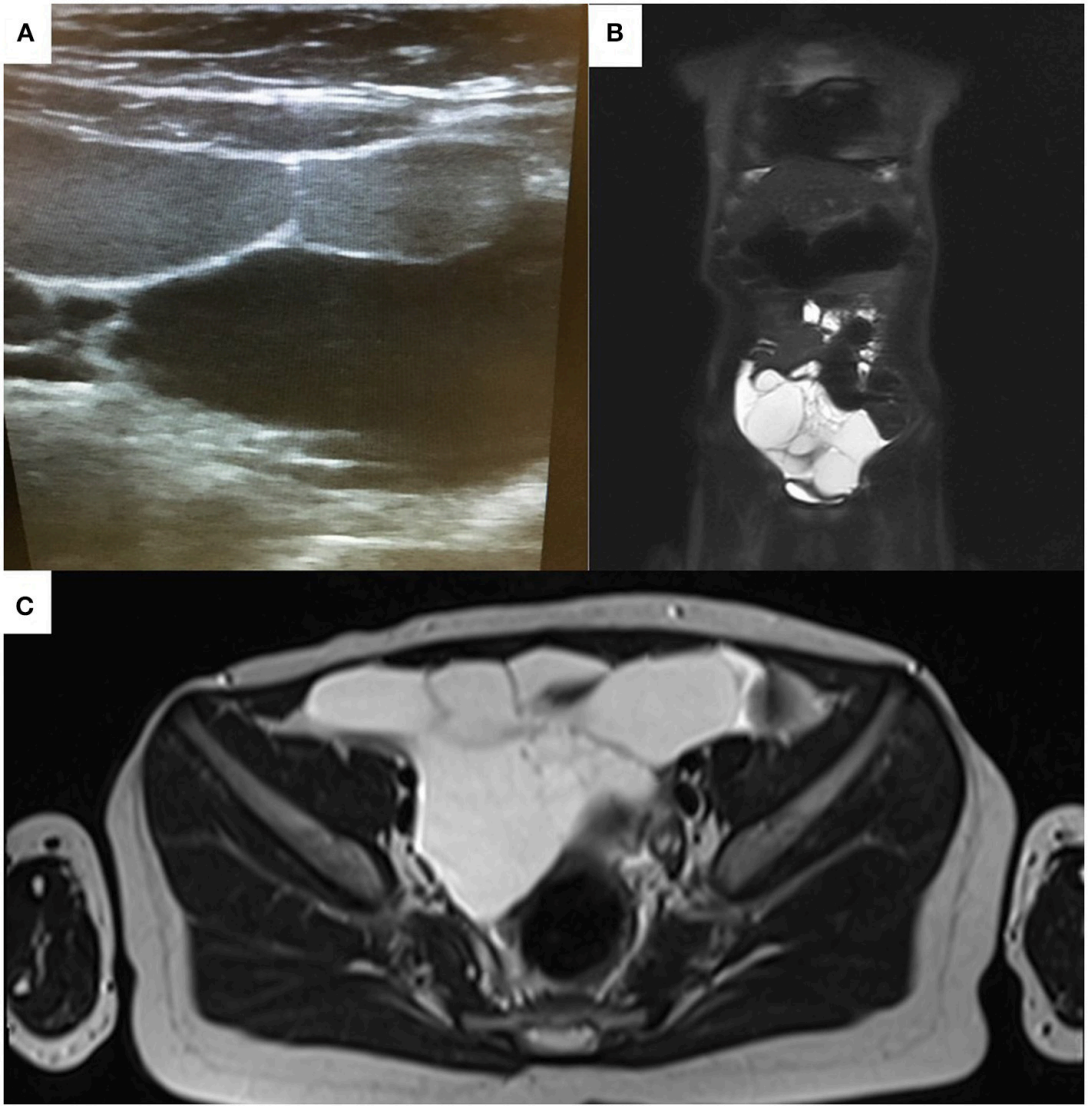
Echographic **(A)** and MRI appearance **(B,C)** of the abdominal tumor mass initially misdiagnosed as acute appendicitis. Note the multicystic appearance **(A)**.

Based on the findings from initial clinical evaluation, the pediatrician recommended an ultrasound. The radiologist described an intraabdominal, multilocular cystic mass with liquid inside (Figure 1A), and subsequently recommended an MRI to certify the diagnosis. The MRI confirmed the presence of the liquid filled multicystic structure with hypersignal in ponderate sequences T2 and T2 with fat suppression. The lesion had a multilocular aspect and a moderate contrast snuff at the level of the walls following contrast substance administration (Figures 1B,C). These radiologic findings strongly support the diagnosis of intestinal lymphangioma, and tumor removal by surgery was recommended.

**Figure 2:**
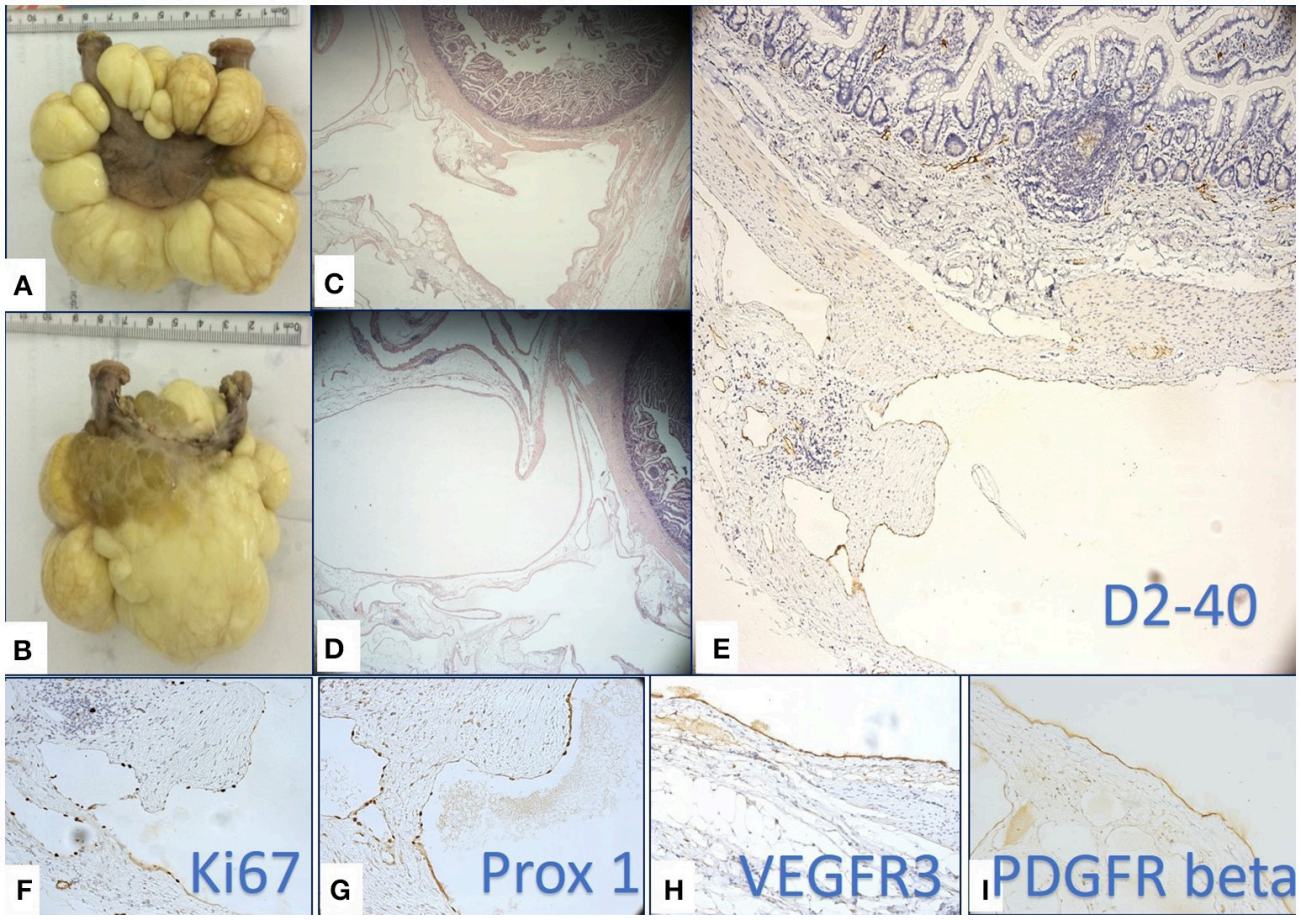
Macroscopic **(A,B)** and microscopic **(B,C)** multilocular, cystic tumor mass removed by surgery. Multiple cysts were filled with yellowish, milky liquid **(B)**. Histopathology revealed several cyst like structure lined by a flat epithelium **(C,D)** proved to be lymphatic endothelium by its positivity for D2-40 **(E)**, Prox-1 **(G)**, and VEGFR-3 **(H)**. High proliferation rate of lymphatic endothelium assessed by Ki67 **(F)** supported the active state of LECs. **(I)** PDGFR-β was intensely positive on LECs of the flat epithelium lining the cystic lesions.

The surgically removed tumor was sent for histopathologic evaluation. Macroscopically, the pathologists identified a 8.9 cm length intestinal fragment, with a 0.5–1.2 cm luminal diameter, that was incorporated in a soft, yellow-translucent mass. When sectioned, there were multiple cysts with a wall thickness that was less that 0.1 cm and a smooth inner and outer surface, filled with a white-milky content (Figures 2A,B).

## Microscopic and immunohistochemical evaluation

Microscopically, the small intestine fragment presented a lymphatic vessel proliferation containing vascular structures of variable size, diffusely disposed in the entire thickness of the wall, predominantly at the level of the serosa (Figures 2C,D). We considered for diagnosis, the immunohistochemical evaluation of the specimen as a useful tool in order to identify the lymphatic origin of the cells that lined the cystic mass and also to evalaute the maturation state and proliferation rate of the LECs. We used D2-40 (specific for LECs), Prox-1 (known to be a marker of immature LECs with a high lymphangiogenic potential), and Ki-67 along with the additional evaluation of VEGFR-3 and PDGFR-β expressions. VEGFR-3 and PDGFR-β are two growth factor receptors against which various targeted therapies are now available. Whereas the implication of VEGFR-3 as a LECs growth factor receptor has already been certified, PDGFR-β remains a controversial lymphangiogenic factor at the time.

D2-40 staining (Figure 2E) certified the lymphatic nature of the cells lining the cystic structures and certified the diagnosis of a mesenteric lymphangioma. The endothelial proliferation evaluation by means of Ki-67 revealed that most of endothelial cells were in proliferating state. The high proliferative state of LECs were specific for cystic spaces of lymphangioma compared with the well-known extremely low LECs proliferation usually observed in the normal lymphatic vessels (Figure 2F). The high LECs proliferation index raised our suspicion of a certain immaturity grade, thus determining us to apply Prox-1 staining. Prox-1 is known as a transcriptional factor that is expressed in the immature LECs in the early stages of lymphatic differentiation from the venous endothelial cells. We observed that the density of Prox-1 nuclear expression (Figure 2G) proved to be similar to the density of Ki-67 nuclear expression, aspects that have strengthened the hypothesis that LECs lining the multicystic lesions are very active, without being completely differentiated. Further examination of the growth factors demonstrated an intense reaction of LECs for VEGFR-3 (Figure 2H) and PDGFR-β (Figure 2I), thus suggesting the fact that lymphangiomas may benefit from a future therapeutic option, namely an anti-VEGFR-3 and anti-PDGFR-β targeted therapeutic approach.

The patient had a favorable postsurgical evolution, without presenting any complications or recurrences in the past year. The patient was evaluated from a genetic point of view and did not present any genetic abnormalities that could certify the occurrence of this lesion.

## Discussions

We presented here a case of a multicystic mesenteric lymphangioma, an extremely rare and versatile vascular entity in children. The evaluation of such lymphangiomas in prior case reports has been similar to our own work, with focus given to LECs that line the multicystic lesions.

The normal genetic profile of the patient challenged us to critically discuss other hypotheses regarding the origin of lymphangiomas, the LECs maturation status, the role of Prox-1 in lymphangioma pathogenesis, as well as the review of the literature considering the role of VEGFR-3 and PDGFR-β in such lesions. In the future, these two receptors will most probably be looked upon as potential specific therapeutic targets for recurrent lymphangioma treatment.

Our results demonstrated the existence of active and immature LECs considering that most of them expressed Prox-1. Until now, the use of Prox-1 in multicystic mesenteric lymphangiomas is sparse in routine practice and this limited use of such marker may explain Prox-1 low impact on therapeutic management of patients diagnosed with lymphangioma (14). But Prox-1 role in lymphangioma development must not be neglected because it highlights a continous potential of endothelial cells specification through lymphatic lineage closely related to endothelial immature state and a high risk of lymphangioma recurrences.

Prox-1 is expressed in the first stages of lymphatic differentiation (15). Prox-1 expression is absent at the level of the mature lymphatic endothelium. In reference to our examined case, considering the high proliferation index observed on lymphatic endothelium belonging to lymphangioma, we may conclude that this vascular malformation has an active state containing activated LECs that may represent a source of cells that may be potentially responsible for the described, and indeed sparse recurrences of lymphangiomas. For this reason, we suggest using Prox-1/Ki-67 combination staining as part of the routine evaluation of lymphangiomas. The expression of these two markers may be regarded as a suggestive prognostic factor for tumor recurrence. The expression of these two markers may possibly be regarded as a suggestive prognostic factor for recurrences.

In 2015, Wu et al. characterized LECs isolated from patients presenting lymphatic malformations as possessing a CD133 positive profile. *In vitro* conditions showed that these CD133 positive cells, having the identity of progenitor cells, proved themselves to be multipotent, and capable of differentiating into adipose cells, bone cells, smooth muscle cells, and LECs. The CD133 negative cells that were isolated from lymphatic malformations did not express stem cell markers, however, they were characterized by an increased Podoplanin, VEGFR-3, and Prox-1 expression. These data are in concordance with the results obtained by us using immunohistochemical assay. The above mentioned authors conclude that LECs isolated from human lymphatic malformations are characterized by an active status and possess a lymphatic progenitor profile that altogether, could contribute to the clinically refractory behavior pattern that is associated with lymphatic malformations (16). Scattered data have reported that cutaneous LECs and lymphangiomas have a relatively identical molecular pattern by expressing Podoplanin and Prox-1.

VEGFR-3 is less studied than Prox-1, but plays a major role in LEC differentiation. VEGFR-3 is overexpressed in LECs belonging to lymphangiomas. Norgall et al. (12) concluded that VEGFR-3 and−2 overexpressions in lymphangiomas contribute to the etiology of lymphangiomas. Currently, VEGFR-3 is not applied in the routine evaluation of human lymphangiomas. Itakura et al. (17) studied the expressions of VEGF-C and VEGFR-3 on a group of 114 lymphangiomas with different locations and concluded that VEGFR-3 is expressed in 50–72% of lymphangiomas with different locations, the expression rate being evaluated at 70% in case of intraabdominal lymphangiomas. Prox-1 and VEGFR-3 co-expression suggests the use of an individualized therapy in lymphangiomas, especially in recurrences. For the moment, there is no available anti-VEGFR-3 targeted therapy that can be applicable in clinical practice.

Few papers reported lymphangioma treatment using an intralesional injection of a steroid, bleomicin, and bevacizumab mixture; the duration of these therapeutic procedures was reported to be up to 1 year long and at 3 years follow-up no recurrences were detected (18). The intralesional therapy with a mixture of sclerosing agents and corticosteroids is indicated to superficially distributed lymphangiomas, that can be directly approached. However, this therapy is impossible in case of lymphangiomas presenting in a mesenteric location. Due to these aspects, the use of certain alternative targeted therapies represents a novel perspective in the lymphangioma treatment.

In 2004, Esmaeli et al. (13) studied the expressions of PDGFR-α, PDGFR-β, and EGFR in orbital lymphangiomas and demonstrated the presence of a positive PDGFR-β expression in the lymphatic endothelium. This is the only available study in which PDGFR-β is mentioned as a potential therapeutic target in lymphangiomas. Whang et al. mentioned that following intralesional combined therapy, the expressions of PDGFR-α and PDGFR-β have decreased. In the present study, we noticed a positive PDGFR-β expression at the level of the endothelial cells that lined lymphangioma.

A recent study conducted on Prox-1, VEGFR-3, and Podoplanin co-expression demonstrated the phenotypic heterogeneity of Podoplanin positive cells that occurred in myocardial infarction. Interestingly, our study revealed that PDGFR-β and Prox-1 co-expression determines a profibrotic phenotype, a fact that may indirectly explain the development of lymphangiomas through a complex mechanism (19).

We considered this extensive evaluation to be useful due to the rarity of the lesion during childhood, a rarity that resulted in the lack of a molecular analysis and in a limited and controversial therapeutic approach.

## Concluding remarks

We hereby described a case of a multicystic mesenteric infantile lymphangioma with an active, immature character that suggests the possible recurrence due to the presence of a cellular and molecular substrate. Until today, no recurrences have been detected for the present case.

This study looked at markers of immaturity and active replication, that could help determine the prognostic value of these tumors and may guide the need for post surgical surveillance. Given the fact that these tumors can be in locations that are not amenable to surgery, pharmaceutical options that are targeted to the individual are needed. This paper identified some markers that can be used as targets for specific therapies. Our results support a review of the protocols evaluation applied in lymphangiomas.

## Ethics statement

Written informed consent for the publication of this case report and figures was obtained from the parents. This case presentation carefully respects the statements and was carried out in accordance with the Declaration of Helsinki.

## Author contributions

SC was the radiologist who described the lesion by echography and MRI. CP performed surgery for tumor removal. RH gave the microscopic diagnosis and wrote the paper. AJ confirmed histopathology and revised the paper. AC and MR are involved in immunohistochemistry and its interpretation, built the discussions, and critically reviewed the final form of the manuscript.

### Conflict of interest statement

The authors declare that the research was conducted in the absence of any commercial or financial relationships that could be construed as a potential conflict of interest. The reviewer MH and handling Editor declared their shared affiliation.
